# The ethics of genomic medicine: redefining values and norms in the UK and France

**DOI:** 10.1038/s41431-020-00798-2

**Published:** 2021-01-17

**Authors:** Marie Gaille, Ruth Horn, The UK-FR GENE (Genetics and Ethics Network) Consortia, Mark Bale, Catherine Bourgain, James Buchanan, Anne Cambon-Thomsen, Herve Chneiweiss, Angus Clarke, Edward Dove, Xavier Guchet, Nina Hallowell, Angeliki Kerasidou, Anneke Lucassen, Fiona Maleady-Crowe, Jennifer Merchant, Michael Parker, Alexandra Soulier, Carine Vassy

**Affiliations:** 1 SPHERE, Université de Paris-CNRS, Paris, France; 2 The Ethox Centre and Wellcome Centre for Ethics and Humanities, Nuffield Department of Population Health, University of Oxford, England, UK; 3 Genomics England, London, UK; 4 Inserm, Cermes3 (UMR CNRS 8211 - INSERM Unit 988 – EHESS), Paris, France; 5 Nuffield Department of Population Health, University of Oxford, Oxford, UK; 6 CNRS, Inserm, Université Toulouse III Paul Sabatier, Toulouse, France; 7 CNRS, Inserm, Sorbonne University, Paris, France; 8 Institute of Medical Genetics, Cardiff University, Wales, UK; 9 J Kenyon Mason Institute for Medicine, Life Sciences and the Law, University of Edinburgh, Scotland, UK; 10 Université de Technologie de Compiègne, Compiègne, France; 11 Clinical Ethics and Law, University of Southampton, Southampton, UK; 12 Centre d′études et de recherches de sciences administratives et politiques (CERSA), University Paris 2, Paris, France; 13 CNRS, Paris, France; 14 IRIS, Université Sorbonne Paris Nord, Paris, France

**Keywords:** Ethics, Medical genomics, Medical ethics

## Abstract

This paper presents a joint position of the UK-France Genomics and Ethics Network (UK-FR GENE), which has been set up to reflect on the ethical and social issues arising from the integration of genomics into routine clinical care in the UK and France. In 2018, the two countries announced enhanced cooperation between their national strategies, Genomics England and Plan France Médecine Génomique 2025, which offers a unique opportunity to study the impact of genomic medicine and relevant policies in different national contexts. The paper provides first insights into the two national strategies and the norms, values and principles at stake in each country. It discusses the impact of genomic medicine on established relationships and existing regulations, and examines its effects on solidarity and trust in public healthcare systems. Finally, it uses the social contract as an analytical lens to explore and redefine the balance between individual rights and collective duties in the context of genomic medicine. This paper leads to three key observations: (1) despite each country’s strategy being at a different stage of implementation, the two countries face similar ethical issues; (2) each country tries to solve these issues by (re-)defining individual rights and collective duties in its own way; (3) the social contract presents a useful tool to analyse the ways the UK and France address the ethical challenges raised by genomics. This overview lays the groundwork for future in-depth comparison, and drive collaborative research, between the UK and France.

## Introduction

In 2018, the UK and France announced enhanced cooperation between their national strategies, Genomics England (GE) and Plan France Médecine Génomique 2025 (PFMG), to develop genomic medicine [1], offering a unique opportunity to study the ethical and social issues arising from the integration of genomics into routine clinical care in these countries. For this purpose, the two principal authors of this paper initiated the UK-France Genomics and Ethics Network (UK-FR GENE) in 2018, with the aim to bring together researchers from each country to reflect on these issues and develop a joint research agenda. The first UK-FR GENE workshop was held in 2019. Amongst other things, it demonstrated that an in-depth understanding of the impact of genomic policies requires a thorough reflection on the norms, values and principles that are at stake regarding genomic medicine and its implementation.

This paper provides a first insight into some of these norms, values and principles in each country and lays the groundwork for future in-depth comparison between the UK and France. We start with describing the two national strategies and proceed to highlight the impact of and changes brought about by the implementation of genomic medicine regarding a variety of relationships (e.g., between patients and healthcare professionals) and regulations such as laws or professional guidelines (e.g., on privacy and data protection) in each country. Finally, we examine how the integration of genomics into routine care raises questions about how to maintain the solidaristic character [2] of, and public trust in, public healthcare systems. We conclude by reflecting on the usefulness of the social contract as an analytical lens to explore and redefine the balance between individual rights and collective duties in the context of genomic medicine. Despite the different stages of development of the two national strategies, they nevertheless offer an important starting point to compare the state of art, the regulations and arguments that shape the implementation of genomic medicine in each country.

## Implementing genomics into clinical care: two national strategies

GE and PFMGs’ aims are to integrate genomics into routine healthcare in order to improve diagnosis for rare disease and cancer and better target personalised treatment options. In the UK, GE launched the 100,000 Genomes Project (100 kGP) in 2012 with the aim of sequencing 100 k genomes from around 85,000 National Health Service (NHS) patients with rare disease or cancer and this has been delivered since 2014 through 13 NHS Genomic Medicine Centres across England [3]. It is important to mention that several genomes may be sequenced for each patient (e.g., “trio testing” whereby the genomes of both parents are sequenced alongside of a child’s in rare disease, and tumour as well as germline genomes are sequenced in cancer). GE is a private company wholly owned by the UK’s department of health and social care offering participants a potential diagnosis as well as the opportunity to take part in research (see below). Thus the 100 kGP has been presented as a ‘hybrid’ between research and clinical care. When rolled out fully in spring 2021, the NHS Genomic Medicine Services will provide access to genetic and genomic testing across England through the public health sector.

In France, in order to drive the development of genomic medicine, the PFMG aims to sequence 235,000 genomes per year from 2020. Four pilot projects will be put in place to improve diagnosis, prognosis and treatment for cancer, rare diseases (e.g., intellectual disability syndromes) and common diseases (e.g., diabetes), and to improve the interpretation of genetic variants by indexing existing variants in the French population. To reach this aim, a network of 12 sequencing services will be gradually set up across the country, of which only two are set up to date.

One of the main challenges regarding the implementation of genomics in both countries concerns obtaining valid consent in the context of complex and often uncertain genomic information. The 100 kGP has adopted an open-ended consent approach allowing participants, as diagnosis evolves, to decide whether to receive secondary (clinically actionable) findings or not; the list of possible secondary findings are determined (but still not finalised) by the 100 kGP not the participants [4]. This approach has been adapted from various recommendations on diagnostic incidental or secondary findings such as the American College of Medical Genetics and Genomics (ACMG). However, it should be noted that GE is proposing to return information about only a small subset of the genes included in the ACMG list of those loci in which secondary findings will be sought. In 2019, the Joint Committee on Genomics in Medicine published guidelines for consent and confidentiality in clinical practice [5]. The guidance highlighted that the definition of a secondary finding as actionable was too simplistic as ‘actionability’ is defined differently by different groups, and variants have different degrees of certainty as well as risk associated with them. Hence, the guidance emphasises the importance of the clinical context in communication of secondary findings as part of the NHS Genomic Medicine Service. Furthermore, the guidance emphasises the potential implications of genomic findings for family members.

To date, France has not adopted a specific legal framework regarding valid consent to genomic testing, and the current Bioethics Law applies to genomic testing. The civil code, in line with the Oviedo convention (1997, ratified by France in 2011), primarily aims at protecting ‘the integrity of the human species’ (ban on eugenics, article 16-4). In addition, it states that genetic tests can only be undertaken for research or medical purposes (article 16-10). Hence, in the clinical context, consent can only be given for specific conditions in symptomatic patients (e.g., for diagnosis or the guidance of treatment) or asymptomatic patients (e.g. in case of susceptibility or predisposition due to family history) [6]. However, in research, it is possible to return incidental findings that indicate a serious genetic disease, if the participant has given his/her explicit consent for this. In 2019, the PFMG has issued patient information sheets and informed consent templates [7] for both research and clinical care. The revision of the Bioethics Law (Title II, Chapter 2, articles 17–18; Title IV, Chapter 2, articles 8 sq.), to be adopted in 2021, may introduce two major changes regarding genetic and genomic testing. First, tests could be allowed even if the person cannot express her will or is dead, if the aim is prevention or improvement of care for relatives. Second, health professionals could be allowed to reveal incidental findings if the patient consents to it prior to testing, including in the case of prenatal testing (in this case, the above mentioned consent form templates will be adapted accordingly). A list with additional findings to search for, as proposed by the ACMG, is not envisaged. The example of valid consent and the scope of testing in the genomic context demonstrate how new technologies initiate change and a rethinking of existing practices and principles.

## The impact of genomic medicine on various kinds of relationships

### Blurred boundaries between research and clinical care

The implementation of genomics in clinical care and research impacts several relationships, including that between research and clinical care. The need for a strict division between research and clinical care was explicitly stated in the Belmont Report (1969). However, the blurring of these activities has been observed in many areas of biomedicine [8], including the field of genomics [3, 9]. Consequently, the implementation of genomics in a (clinical) research setting raises a number of ethical issues for both researchers and clinicians, such as when should sequencing activity be regarded as research or care [3], how should clinically relevant findings generated during clinical research sequencing be managed and who should be responsible for their management [10]. A lack of clarity about the nature of sequencing activities, whether they are research or care, raises important questions regarding the relationship between researchers (clinicians) and participants (patients), specifically, the extent to which they have a duty to feedback clinically significant research findings to these stakeholders and their family members, who may have been involved in undergoing sequencing for research purposes.

In the UK, attempts have been made to impose some ethical boundaries on sequencing research in order to circumvent these ambiguities. For example, in GE’s 100 kGP, access to whole-genome sequencing to aid their clinical diagnosis/treatment, required patients to consent to their DNA samples being used in research, and also gave them an opportunity to consent to receiving a prescribed, limited list of health-related additional findings. In France, at the time of writing this paper, the issue of communicating additional findings in research and clinical care remains unsettled [11].

Despite the questions raised by the blurred boundaries between research and clinical care in genomics, it is important to acknowledge that the shared aim of both, research and care, is the improvement of health.

### Changing relationships between patients and health professionals

Genomics challenges not only the boundaries between research and clinical care but also the relationship between patients and healthcare professionals. Genomic tests, e.g., for diagnostic or predictive purposes, are complex processes and rely on evolving sets of skills, which can challenge healthcare professionals. Laboratory reports may not always be definitive [12], and the interpretation of results not always straightforward. It has been reported that professionals often are uncomfortable interpreting the clinical significance of the results and communicating these to the patients [13] not least because for some of the detected conditions/genetic predispositions, treatment or prevention may not be available, and patients may have overly optimistic expectations of what genomic testing may reveal [14]. In some cases, patients may have undergone a series of unsuccessful examinations to obtain a diagnosis before they are offered a genomic test, whereas others may have a family history of a genetic condition. As genomics is entering most areas of clinical medicine, more healthcare professionals have to respond to new challenges and are faced with complex genomic information, often with rather limited training in, or exposure to, genomics.

In addition to these challenges and uncertainties, genomic testing also raises questions about professional duties such as the duty to recontact patients if understanding of the implications of a detected variant changes since the time of testing [15]. Furthermore, when a genomic test is performed, there may be unexpected findings that are not only relevant to the patient, but also to their broader family. In such cases, clinicians may become aware of tensions in their duty to care for their patient and respect his/her confidentiality, and their duty to use this genomic information in the care of his/her family [16].

The UK and France have addressed these issues by balancing collective and family responsibilities against individual rights in different ways. For over a decade, French legislation has put a strong emphasis on the rights of relatives to be informed about genetic tests results if they are relevant to them. The law requires either and preferably the patient, or the doctor (according to a specific procedure protecting the confidentiality of the patient) to communicate the relevant information to relatives (Law No. 2011-814, 2011). In England, such a duty has been established as a duty to weigh the interests of relatives in the balance with the interest of maintaining the confidentiality of one person (a recent court ruling confirms this [2020] EWHC 455 (QB)). Often the interests of both patients and family members can be respected without the need to decide in favour of preservation or breach of confidence [17]. Generally, we observe an emphasis in both countries on considering the broader collective, familial or societal, aspect of genomics.

### Public involvement and the democratic sharing of knowledge about genomic medicine

The increasing use of genomics in clinical care and the complexity and challenges raised by the technologies to assess genomics have led to concerns about public understanding and acceptance of genetics and genomics [18]. There have been attempts to encourage a ‘peaceful’ debate about these issues [19], by offering citizens the opportunity to obtain information so that they may assess, and participate in, decisions about health policies and the allocation of resources. However, while public engagement and lay communication about genomic medicine are crucial for broadening this debate, several problems have been identified with this, such as, the oversimplification of new scientific insights, the difficulty of addressing the ‘real public’ [20] rather than specific groups, and the risk of adopting a one-way communication model where experts educate the lay public [21].

Both the British and French governments emphasise the importance of public engagement in the context of genomics. For example, the recent PFMG report addresses the question of how to inform and involve civil society [22] and has charged the National Institute for Health and Medical Research (INSERM) with implementing this. Similarly, Patient and Public involvement is an essential aspect of GE’s strategy to implement genomic medicine. However, one central issue regarding public engagement, in both countries, is how engagement with publics, e.g. knowledge sharing, is conceived beyond the application of the minimal, and somehow rhetorical, principle of ‘you should at least ask’ [23]. One aspect to take into account is that we are in an era of epistemic transition [24]. Education about genomics in life sciences programmes in schools, as already happens in French high schools, could also improve public understanding and involvement in the future.

Another issue is the involvement of members of the public in research by co-opting one or two “public representatives” as members of a project steering group. It is currently unclear how effective such initiatives may be, in terms of the extent to which these individuals can influence the research process, as well as its ethical and legal framing. In order to effectively involve the public, the only way to proceed may be to adopt democratic procedures and tools, however, ‘imperfect, slow, difficult and expensive’ they may be [25]. As we will see in the next section, transitions and changes in the genomic era not only occur at a relational level (e.g., between professionals and patients or science and the public), but also at a regulatory level.

## Changing regulations

### The protection of personal data at European and national levels

At the highest level, human rights law governs a person’s private life, including data about them. For the purpose of this article, we focus on data protection law at a European as well as national level. The General Data Protection Regulation 2016/679 (GDPR) came into force in 2018. It regulates the processing of personal data across the European Union and European Economic Area. At the time of writing, the GDPR still applies in the UK, despite its exit from the EU. The GDPR promotes responsible data processing for the benefit of the common good, while also respecting individuals’ fundamental rights. While it is a binding legislative act it also provides a wide margin of manoeuvre for EU Member States, including for the processing of ‘sensitive data’ such as genetic data [26].

In the UK, while there is no specific law governing the processing of genetic data, the Data Protection Act 2018 gives effect to the GDPR. This stipulates that processing ‘special category data’ for scientific research is permitted only if it does not cause harm to the data subject, is approved by a research ethics committee, and is in the public interest. In France, sensitive data have been protected by the ‘Law on Data Processing, Data Files and Individual Liberties’, since 1978 and by the French Bioethics Laws of 1994. Any processing of sensitive data in the public sector must be authorised by the National Commission on Informatics and Liberty (Law no 2018-493 of 20 June 2018). Consequently, any processing of sensitive data requires informed consent of the concerned person (or another valid legal basis), prohibits any commercial use of this information, and ensures the right to privacy (i.e., only the health care professional in charge has access to genetic information). Any violation of the processing of sensitive data is sanctioned by the criminal code (articles 226-25–226-30).

### Balancing privacy and data sharing

The term ‘privacy’ is not mentioned per se in the GDPR (nor is the term ‘private life’). Data protection and privacy are related but distinct concepts. Privacy, at least in its informational dimension, is a state of affairs whereby data relating to a person are in a state of non-access. It embodies a broad range of rights and values, such as the right to be left alone, intimacy, seclusion, and personhood. Data protection is a set of legal rules protecting the rights, freedoms, and interests of individuals whose personal data are processed. The definitive objective of data protection is to ensure fairness in the processing of data and, to some extent, fairness in the outcomes of such processing. The aims of data protection, then, are broader than simply privacy protection, but at the same time, data protection is a crucial tool to ensure privacy. The framing of privacy protections in other EU texts and conventions mentioned above present a political–philosophical approach that should inform laws, be they on the EU level or the member-state level. In addition, the inclusion of genetic information within the category of sensitive data should be noted [27].

Whereas privacy (and data protection) are an expression of our individual liberty, and hence constitute core values in European society, they are not absolute and must be balanced with other values such as the promotion of (individual and public) health. As the GDPR and its national adaptations demonstrate, it may be appropriate to share genomic and health-related data that is potentially (re-)identifiable with certain authorities and institutions. In addition to this, the GDPR introduces a new ‘right to data portability’ (article 20) enabling individuals to receive their personal data, e.g., genomic data, which they have provided to a controller (such as a research organisation or hospital), in a ‘structured, commonly used and machine-readable format’, and to transmit those data to another data controller, e.g., for secondary or otherwise further analyses. This raises important questions of how to implement this right without creating or exacerbating data security risks.

Since the launch of the Human Genome Project in 1990, genomic research has been a pioneering field in the development of data sharing practices and policies. Today, large funding bodies require the sharing of sequence data to be considered in all funding applications in genomics. The rationale for such a change is that data produced by publicly funded bodies should be available as soon and as widely as possible in the research community to enhance scientific endeavours and benefit the public. However, data sharing in genomics as well as in other scientific fields raises practical and ethical issues, since sharing practices may complicate responsibilities towards participants and make it difficult for data creators and curators to be acknowledged within current systems of scientific evaluations.

In the PFMG, data sharing practices are recognised in principle. Their implementation and conformity with the GDPR will be discussed as part of the next strategy phase, when data will be produced. GE has already adopted a ‘reading library’ model, where the data are stored in the National Genomic Research Library and is accessed by researchers via a Trusted Research Environment. This is a comprehensive resource allowing approved researchers to securely access de-identified genomic and other associated health data within the Library. Anyone accessing the 100 K GP dataset—including GE staff, service providers, academic or commercial researchers—must apply to an independent Access Review Committee to access de-identified data and demonstrate the scientific contribution of their research or its potential benefit to the participants. The Committee is made up of senior independent experts and an equal number of participants drawn from the GE Participants Panel. The Committee also monitors the progress of research and the opportunities for rapid implementation of findings to benefit patients. The limitation of privacy protection in the UK and France only seems to be justified when these individual rights are outbalanced by collective benefits and a commitment to the common good.

## Towards a new social contract

### Trust and solidarity

Solidarity is the founding principle of publicly funded healthcare systems, it occupies an important place in the development of healthcare policies in Europe, and has underpinned the social contract between healthcare systems and members of society since the late 19th century. Solidaristic healthcare systems are based on ‘a feeling of responsibility […] togetherness and commitment to the common good’ [28] which provide the basis for public trust [29]. Although the British and French public healthcare systems are among the most trusted and respected public institutions in these countries, funding cuts have led to a decrease in trust in recent years [30, 31]. Some governments seem to try to address the funding crisis in healthcare by focusing on new health technologies, such as data-driven sciences, that promise economic growth and on partnerships with private companies [32].

The two initiatives, GE and PFMG, are examples of national strategies that are centred on genomics, with the aim of growing new industries, forging collaborations between the public and private sector and demonstrating the economic value of health data. However, profit-driven collaborations have the potential to challenge the solidaristic character—i.e., the focus on the common good of public actors (or, as is the case with GE, government owned companies)—of these healthcare systems. This is particularly important when these collaborations involve patient data as it raises concerns regarding confidentiality and privacy, but also trust among the public. For example, in 2016, heavy criticism arose after the UK’s data protection watchdog ruled that a deal to share 1.6 million NHS patient records with the Google-owned artificial intelligence company DeepMind ‘failed to comply with data protection law’. Patients had not been informed that the company would be given access to their data. To address the concerns and provide transparency, DeepMind published all its contracts. Yet, in 2019, new concerns were raised when a number of NHS trusts transferred these contracts to Google Health, who refused to make the new contracts transparent [33], failing to obtain public approval, i.e., a ‘social licence’ [34].

Despite the difficulties raised by public–private partnerships, some healthcare systems, such as NHS England, may decide to enter commercial partnerships to gain early access to technology or because they hope for financial advantages. The question arises then as to whether it is possible to build trustworthy public–private sector partnerships that preserve the solidaristic character of public institutions and actors [35]. Arguably, if public healthcare systems want to maintain their solidaristic character and foster public trust, a solidarity grounded partnership model with private companies may offer a solution. Such a model needs to serve collective interests by, for example, securing preferential access to goods and services, providing health benefits, and monitoring data access (e.g. GE’s ‘research library’ model).

## What is the ‘value’ of genomics?

Trust in genomics not only concerns trust in institution, but also trust in the ‘value’ of the technology, i.e., its clinical utility, benefits, efficiency and quality, and in the way it is set-up to be ‘valuable’, hence trustworthy. The use of genomics in clinical care may lead to diagnoses and treatments that are more detailed and focused on a single patient, and which may result in improved health outcomes. Commonly, when a new healthcare intervention is integrated into clinical practice, careful cost-benefit analyses are undertaken to determine its ‘value’ i.e. to weigh its health benefits against the costs [36]. The criteria used to determine this value vary between countries. Alternatively, value can be determined by asking people directly about their health preferences and perceived benefits of a new intervention, using discrete choice experiments (DCE) [37]. Genomic tests are currently entering clinical practice in the UK and France without this evidence and little data on the health economic value of genomics exist in the literature. A recent systematic review reported that the current evidence base on the cost-effectiveness of genome sequencing is insufficient to support widespread use of these tests in clinical practice [38]. In addition, the amount of money patients and/or healthcare professionals would be willing to spend on genomic tests, and the general benefits attributed to these, varies widely, according to the DCE literature [39]. Finally, although it is often stated that genomic data provides wider benefits for national economies [40], no studies have evaluated this broader economic value to date.

Another aspect to be considered in the determination of the ‘value’ of genomics is data quality. In order to fulfil the promises made by genomics, data must meet strict quality, standardisation and ethical requirements. These requirements ensure comparability and exchange of data to support collaborations, transparency and reproducibility and ultimately scientific advances [41].

In order to promote public trust and support of genomics, the ‘value’ of genomic data for science, public health and the economy must be carefully examined, and thorough studies are needed to ensure that resources are allocated efficiently and to the benefit of the common good.

### The social contract in the light of genomic technologies

Genomics challenges broadly accepted individual-focused principles (e.g., valid consent or privacy) and requires these to be redefined in the light of, and balanced with, collective values and benefits. Against this background, bioethicists and policy-makers have begun to discuss the need to rethink the ‘social contract’ between medicine/science and society, away from exclusively individual-focused solutions [42, 43]. The concept of a social contract has been developed by political philosophers (e.g., Locke, Rousseau or Rawls) to describe a set of formal and informal norms and rules that lay out expectations of the rights and duties of citizens [44].

In England, a country with an individualistic tradition, yet also a solidaristic healthcare system, public institutions and documents suggest there is a need for a ‘new social contract for genomic medicine’, and greater focus on the collective dimensions of genomics i.e. the importance of community participation, public interest and trust [43, 45, 46]. For example, GE has called upon the public’s civic duty to participate in the 100 kGP as a contribution to the common good and an expression of solidarity [47]. Yet, since the value of genomics, and hence its contribution to the common good, has not yet clearly been defined, the question arises as to whether the call for solidarity is merely rhetorical.

However, beyond the mobilisation of the idea of the social contract at a policy level, the social contract can provide a useful lens to analyse, at a theoretical level, the ethical challenges raised by genomics. These challenges arise because genomics questions the appropriateness of well-established individual-focused principles of 20th century bioethics such as valid consent, confidentiality or privacy, which have come to shape the doctor–patient relationship, practices and public trust in medicine in many Western countries [48]. Employing the social contract as an analytical lens allows us to explore how each society responds to the challenges posed by genomics, i.e. how it (re-)defines and balances collective and individual rights and duties, and values associated with these.

Presently, the UK follows an approach that prioritises the interests of the individual, and respect for confidentiality and privacy is paramount, as demonstrated by the ‘research library’ model adopted by GE and the legal disputes about the recognition of the rights of the family with regard to genomic information. On the contrary, in France, even though policy debates and legal regulations of genomics also focus on individual rights [49], high value is placed on familial interests that go beyond those of the individual such as is the case with regard to familial access to genomic information. These are just some indications of how each country has begun to negotiate and redefine collective and individual responsibilities and rights in the context of genomics.

## Conclusions

This paper provides an overview of some of the ethical issues raised by the UK and French national strategies to establish genomic medicine as part of routine healthcare. This overview is part of a broader research ambition to offer in-depth understanding of the impact of genomic medicine and relevant policies in these two countries.

Our paper leads to three key observations: First, it emerges that the two countries face similar ethical issues (e.g., blurred boundaries between research and clinical care, securing valid consent in the context of complex and uncertain information), even though their national strategies are not at the same stage of implementation. A second observation is that each country tries to solve these issues by balancing individual and collective interests differently. For example, the limitation of privacy rights is justified in both countries only if this serves the common good. The GDPR is an example of this and shows how regulations (e.g., laws or professional guidelines) express commonly accepted values and frame professional, individual and public decisions. Our third observation is that the concept of the social contract presents a useful tool to analyse the ways the UK and France address the ethical challenges raised by genomics. As highlighted, the collective aspects of genomic information question the appropriateness of individual-focused ethical principles (e.g. valid consent, confidentiality or privacy) that have been established in response to advances in medicine and changing social attitudes in the 20th century. This raises similar ethical issues across countries that promote individual rights and liberties, such as the UK and France, and requires each country, in its own way, to redefine individual rights and collective duties.

These three observations are relevant for further analysis of the ethical issues, yet they do not lead to the conclusion that the UK and France address these issues similarly. Our discussion identifies significant differences between the two countries, especially with regard to individual versus family rights, approaches adopted towards consent or public engagement models, and the role of private companies in the public healthcare system. These and other aspects should be further investigated. The aim of UK-FR GENE is to drive collaborative research and a series of workshops to generate an in-depth understanding of similarities and differences in the ways each country addresses the ethical issues identified.
